# Sperm DNA methylation alterations from cannabis extract exposure are evident in offspring

**DOI:** 10.1186/s13072-022-00466-3

**Published:** 2022-09-10

**Authors:** Rose Schrott, Jennifer L. Modliszewski, Andrew B. Hawkey, Carole Grenier, Zade Holloway, Janequia Evans, Erica Pippen, David L. Corcoran, Edward D. Levin, Susan K. Murphy

**Affiliations:** 1 Duke University Program in Environmental Health, Nicholas School of the Environment, Duke University, Duke, PO Box 90534, Durham, NC 27701 USA; 2 Department of Obstetrics and Gynecology, Division of Reproductive Sciences, Duke University Medical Center, Durham, NC USA; 3 Duke Center for Genomic and Computational Biology, Duke University Medical Center, Durham, NC USA; 4 Department of Psychiatry and Behavioral Sciences, Duke University Medical Center, Durham, NC USA; 5 Department of Genetics, University of North Carolina, Chapel Hill, Durham, NC USA

**Keywords:** *DNA methylation*, *Sperm*, *Neurodevelopment*, *Cannabis*, *Epigenetics*

## Abstract

**Background:**

Cannabis legalization is expanding and men are the predominant users. We have limited knowledge about how cannabis impacts sperm and whether the effects are heritable.

**Results:**

Whole genome bisulfite sequencing (WGBS) data were generated for sperm of rats exposed to: (1) cannabis extract (CE) for 28 days, then 56 days of vehicle only (~ one spermatogenic cycle); (2) vehicle for 56 days, then 28 days of CE; or (3) vehicle only. Males were then mated with drug-naïve females to produce F1 offspring from which heart, brain, and sperm tissues underwent analyses. There were 3321 nominally significant differentially methylated CpGs in F0 sperm identified via WGBS with select methylation changes validated via bisulfite pyrosequencing. Significant methylation changes validated in F0 sperm of the exposed males at the gene 2-Phosphoxylose Phosphatase 1 (*Pxylp1*) were also detectable in their F1 sperm but not in controls. Changes validated in exposed F0 sperm at Metastasis Suppressor 1-Like Protein (*Mtss1l*) were also present in F1 hippocampal and nucleus accumbens (NAc) of the exposed group compared to controls. For *Mtss1l*, a significant sex-specific relationship between DNA methylation and gene expression was demonstrated in the F1 NAc. Phenotypically, rats born to CSE-exposed fathers exhibited significant cardiomegaly relative to those born to control fathers.

**Conclusions:**

This is the first characterization of the effect of cannabis exposure on the entirety of the rat sperm methylome. We identified CE-associated methylation changes across the sperm methylome, some of which persisted despite a “washout” period. Select methylation changes validated via bisulfite pyrosequencing, and genes associated with methylation changes were involved in early developmental processes. Preconception CE exposure is associated with detectable changes in offspring DNA methylation that are functionally related to changes in gene expression and cardiomegaly.

These results support that paternal preconception exposure to cannabis can influence offspring outcomes.

**Supplementary Information:**

The online version contains supplementary material available at 10.1186/s13072-022-00466-3.

## Background

Studies of paternal preconception exposures have highlighted the important role that a father’s exposure history plays in influencing his offspring’s health outcomes [1, 2]. Such work has focused on the effects of exposures on the sperm epigenome, as gametic epigenetic modifications are a plausible mechanism through which exposures can elicit heritable effects. Impacts on sperm DNA methylation have been assessed most often, as DNA methylation is a stable, covalent modification that can influence the way a gene is expressed in a potentially heritable manner [3, 4]. DNA methylation is an important component of spermatogenesis as the sperm methylome continues to undergo reprogramming throughout the maturation process [5]. While this dynamic state is necessary for spermatogenesis [5], the inherent plasticity of the methylome during this critical developmental window might increase the likelihood that an environmental exposure could interfere with this process and have potentially harmful effects [6].

The ability of C*annabis sativa* to heritably impact the sperm epigenome is gaining interest as cannabis legalization is expanding across the United States (U.S.). Currently, 18 states and Washington D.C. have legalized recreational cannabis use, with additional states poised to follow suit. Epidemiologic studies report that cannabis use during pregnancy is associated with adverse outcomes in offspring, including neurodevelopmental, musculoskeletal, and cardiovascular defects [7–12], but little is known about effects of paternal preconception use.

Work by others has shown that exposure of both male and female rats to THC elicits neurobehavioral and physiological changes in their offspring, inclusive of DNA methylation changes in the brain (e.g., see [13–17]). We previously reported that cannabis use by men is associated with methylation changes in their sperm as compared to controls [18], and further that these changes are partially ameliorated following drug cessation [19]. Using a rat model, we found significant changes to sperm DNA methylation in THC exposed animals relative to controls and demonstrated initial support for the heritable effects of THC exposure in rats at autism candidate gene *Dlgap2 *[20]. Subsequent related studies demonstrated synaptic cholinergic deficits, abnormal locomotor activity, and impaired cognitive functions in rats born to THC- or cannabis extract (CE)-exposed fathers as compared to controls [21–24].

These studies provide compelling data about long-term impacts of paternal THC and CE exposures on neurodevelopmental and behavioral outcomes. There remains a need to characterize the effect of cannabis on the entirety of the sperm DNA methylome to fully understand what epigenetic changes might be transmitted to offspring, and how they might contribute to the previously reported phenotypes. Additionally, more work is needed to understand whether a “washout” period would resolve the effects of drug exposure, which would have important public health implications.

In the present study, we examined the persistence of epigenome-wide changes in sperm DNA. We performed whole genome bisulfite sequencing (WGBS) using sperm from three groups of rats. The first was exposed to CE for 28 days, followed by 56 days (~ one spermatogenic cycle) of vehicle only; the second was treated with vehicle for 56 days followed by 28 days of CE; and the third was a control group exposed to vehicle only. Our objectives were to quantify the genome-wide methylation changes in the sperm of the exposed versus control groups and determine whether a ‘washout’ period following exposure mitigates the effects; to validate select WGBS results using bisulfite pyrosequencing; to determine whether any methylation changes present in the F0 sperm were similarly present in F1 offspring tissues, and if so, if they were associated with changes in gene expression; and to determine in offspring of exposed fathers if there were any phenotypic effects that resembled the teratological trends observed in population-based studies.

## Results

### CE exposure timing impacts magnitude of methylation change

**Fig. 1:**
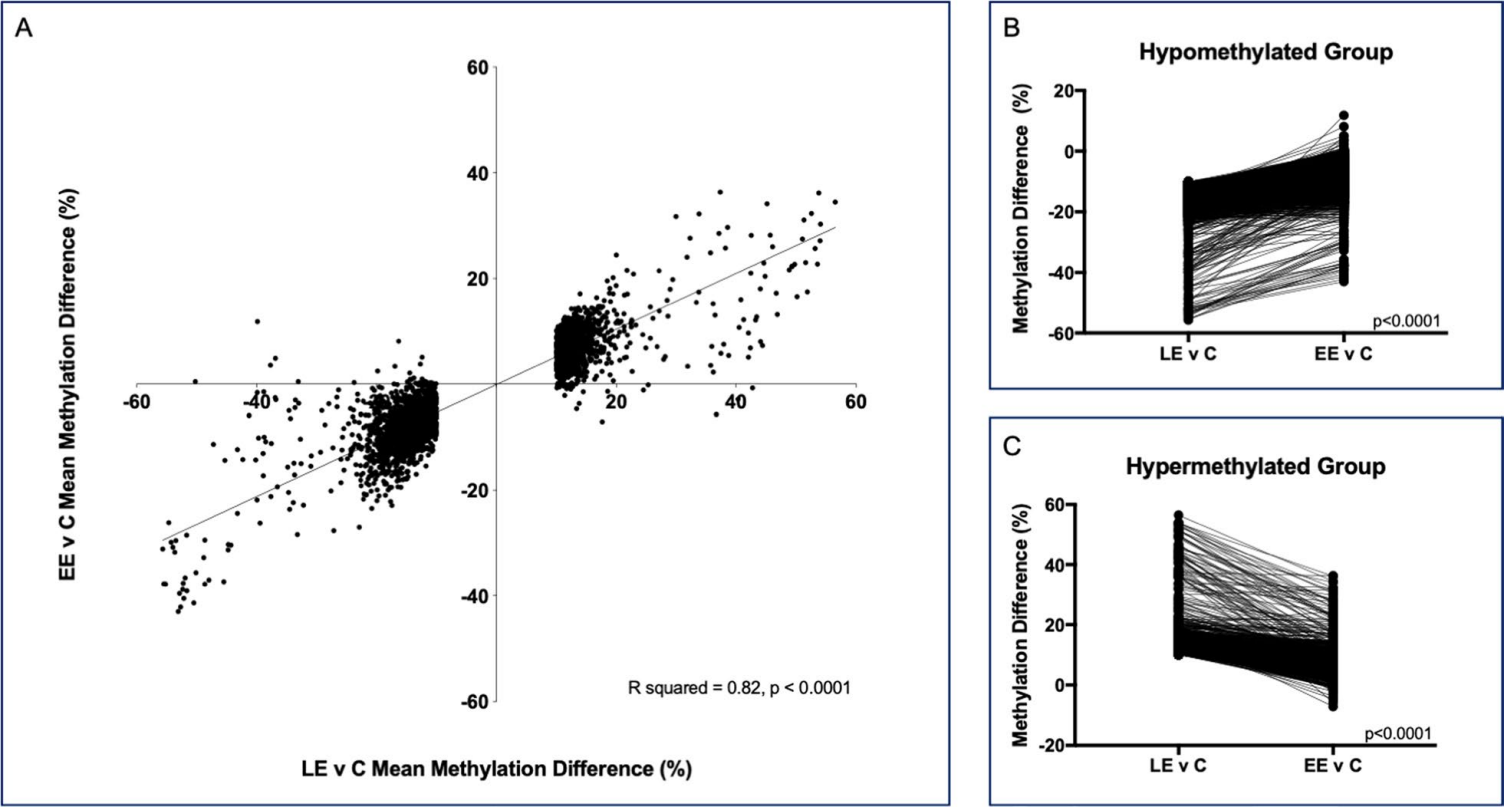
The impact of exposure timing on DNA methylation. **A** Methylation differences at the top 3321 dmCpG sites between the late exposed (LE) and control animals are plotted on the x-axis. Methylation differences between early exposed (EE) and control animals at those same CpG sites are plotted on the y-axis. Negative values represent hypomethylation relative to controls, while positive values represent hypermethylation relative to controls. Linear regression shows significant agreement between the direction of methylation change across the exposure groups (*p* < 0.0001, R2 = 0.82). Of the top 3321 dmCpG sites between LE and control animals, we separated those that were hypomethylated relative to controls (**B**) from those that were hypermethylated relative to controls **(C)**. For **B** and **C**, the methylation difference at each CpG site is plotted on the y-axis. The x-axis represents the methylation difference at a specific CpG site between the LE and control animals and the methylation difference at that same CpG site between the EE and control animals, connected by the solid line. This demonstrates that regardless of the direction of methylation change, the magnitude of the mean methylation difference at each CpG site is significantly reduced between EE and control sperm relative to the difference present between the LE and control sperm (*p* < 0.0001, Bonferroni corrected)

The mean library insert size for the WGBS was 195.9 bp (SD 8.3); mean coverage was 20.2 (2.1); and the mean GC content was 22.7 (0.2). We compared methylation differences from the late exposed (LE) versus control dataset to the methylation differences in the early exposed (EE) versus control dataset to determine the role of the 56-day wash-out period on sperm DNA methylation changes. No CpG sites remained significant following conservative Bonferroni correction, so we imposed a methylation difference threshold on the top 10 K sites, retaining only those with a > 10% methylation difference in the LE relative to control datasets. This resulted in 3321 nominally significantly differentially methylated CpG (dmCpG) sites. We then analyzed methylation at those same 3321 dmCpG sites in the EE relative to control dataset. Regardless of the exposure timing, the direction of methylation change at these sites is largely the same. Linear regression of the data showed significant correlation between LE and EE mean methylation differences relative to controls (Fig. 1A, *p * < 0.0001, R^2^ = 0.82).

### Methylation changes are diminished following cannabis abstinence

We separated the data based on the direction of methylation change present at the 3321 CpG sites from the LE compared to control dataset. This resulted in 1844 hypomethylated CpG sites and 1477 hypermethylated CpG sites in the exposed animals relative to the controls. Comparison of the methylation change at each CpG site shows that the magnitude of the methylation difference relative to controls is greater in the LE sperm than it is at the same CpG sites for the EE sperm for both the hypomethylated (Fig. 1B) and hypermethylated CpGs (Fig. 1C).

The mean methylation difference relative to controls across the 1844 hypomethylated CpG sites was 15.5% in the LE dataset which contrasts significantly with the 8.5% difference at the same CpG sites in the EE dataset (Fig. 1B, *p* < 0.0001). Similarly, there was a 13.9% mean methylation difference relative to controls across the 1477 hypermethylated CpG sites in the LE dataset as compared to a 7.5% difference at the same CpG sites in the EE dataset (Fig. 1C, *p* < 0.0001). These differences remained significant following Bonferroni correction for the number of CpG sites analyzed.

### Methylation changes occur at genes involved in development

Ingenuity Pathway Analysis (IPA) software was used to interrogate the genes associated with the top dmCpGs from both datasets. Briefly, for those CpG sites with a greater than 10% methylation difference relative to controls, an unadjusted p-value threshold of 5.0X10^−5^ was implemented in both the LE and EE datasets to restrict the number to those with the greatest significance. This resulted in a list of 744 CpG sites from the LE dataset and a distinct list of 317 CpG sites from the EE dataset. Notably, only five CpG sites were in common between these two lists. IPA recognized and included 492 and 203 LE and EE dataset genes, respectively, in the downstream analyses.

**Fig. 2:**
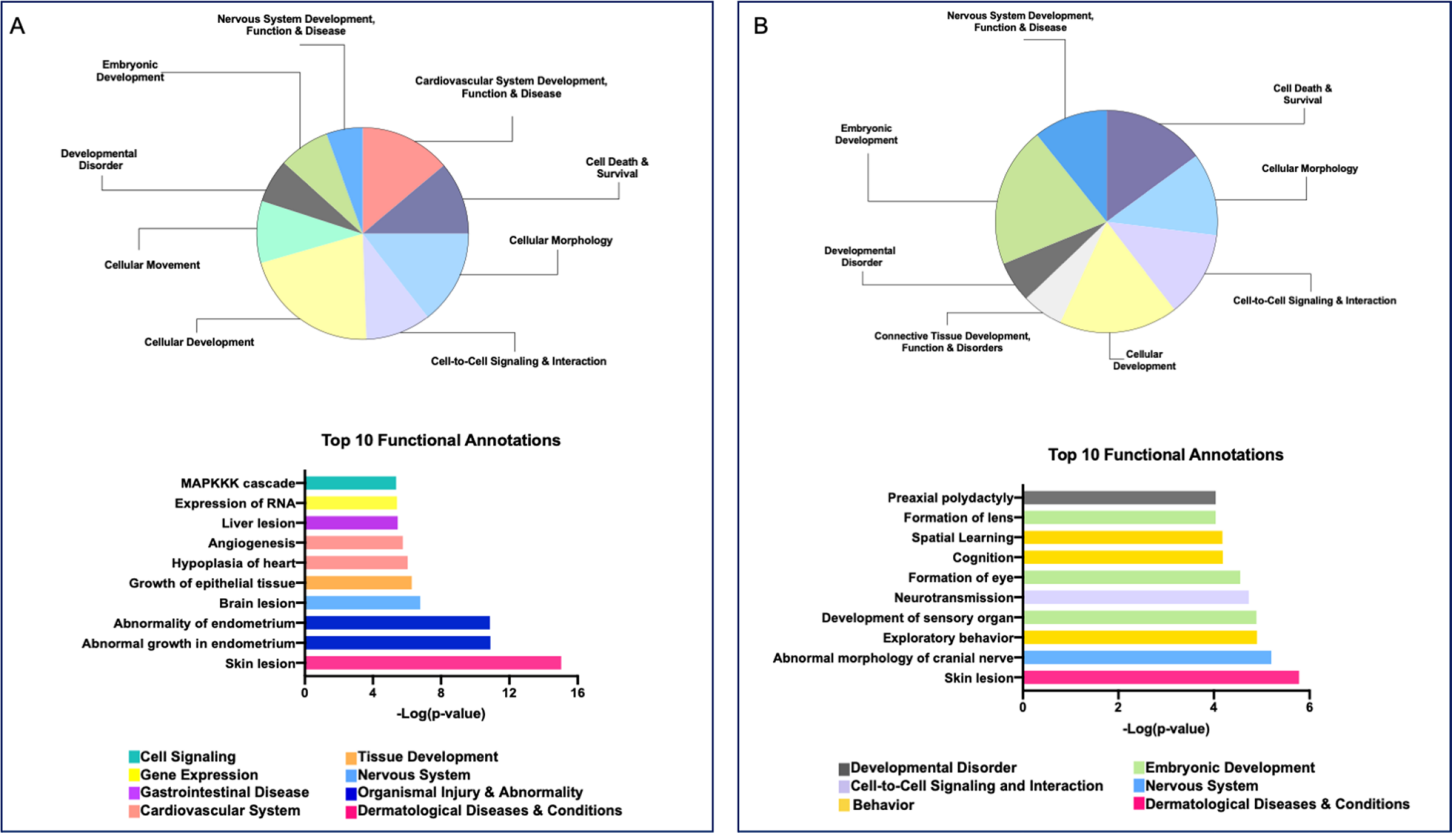
Top IPA disease and functions. (A-top) Pie chart of disease and function categories that have 10 or more associated terms from the LE dataset. (A-bottom) graph of the top ten most significant disease or function annotations from the LE dataset. (B-top) Pie chart of disease and function categories that have 10 or more associated terms from the EE dataset. (B-bottom) Graph of the top ten most significant disease or function annotations from the EE dataset. For both A and B bottom graphs, the Y-axis represents the annotation, and the x-axis is the -log of the p-value. The colors correspond to the category to which this disease or function was annotated (represented by the color legend on the bottom)

We began with the IPA-generated list of 500 significant functions categories and their associated annotations for the LE dataset. Given the overrepresentation of background information associated with cancer in the IPA Knowledge Base, we removed categories associated with cancer from our analysis, as has been reported previously [19]. We also removed terms associated with only a single gene. This left 289 categories and their associated disease or function annotations. We then analyzed the data by first looking at categories with ten or more annotations (Fig. 2A, top). These categories included “cardiovascular system development, function, and disease”, “embryonic development”, “nervous system development, function, and disease”, and “developmental disorder”. We also identified the top ten most significant disease or function annotations (Fig. 2A, bottom).

We next focused on the significant dmCpG-associated genes following the wash-out period in the EE dataset. The workflow for the IPA analysis for the EE dataset is as described above, and resulted in 319 categories remaining. Categories with ten or more diseases or functions annotated (Fig. 2B, top) included “connective tissue development, function and disorders”, “developmental disorder”, “embryonic development”, and “nervous system development, function, and disease”. The top ten most significant disease or function annotations included neurotransmission, cognition, spatial learning, and exploratory behavior (Fig. 2B, bottom).

### Validation of select WGBS results

From both the LE and EE datasets, distinct genes of interest were identified for independent validation. Sites were selected based on > 10% methylation difference, having ≥ 2 significantly dmCpG sites within a 100 bp region, the associated gene having a role in early life developmental processes, and the CpG site being located within the body of the gene. The following genes were selected: Homeobox Protein Hox-B9 (*Hoxb9*)*,* Methyltransferase Like 11B (*Mettl11b*)*,* Slit Guidance Ligand 2 (*Slit2*), LDL Receptor Related Protein 1 (*Lrp1*)*,* Citron Rho-Interacting Serine/Threonine Kinase (*Cit*)*,* Synaptotagmin (*Syt17*)*,* Synaptonemal Complex Protein 3 (*Sycp3*)*,* Gamma-Aminobutyric Acid Type A Receptor Subunit Beta2 (*Gabrb2*)*,* Oncostatin M (*Osm*)*,* 2-Phosphoxylose Phosphatase 1 (*Pxylp1*), Glutamate Ionotropic Receptor NMDA Type Subunit 2A (*Grin2a*)*,* Synapsin III (*Syn3*)*,* Netrin G1 (*Ntng1*)*,* and Metastasis Suppressor 1-Like Protein 1 (*Mtss1l*)*.* We confirmed performance of each of the pyrosequencing assays using defined mixtures of unmethylated and methylated DNAs (R^2^ = 0.85–0.99, *p* < 0.0001–0.025, Additional file 1: Fig. S1)*.*

There were no significant methylation changes found in the sperm of the LE rats compared to the sperm of the control rats at *Hoxb9, Mettl11b, Slit2, Lrp1, Cit*, *Grin2a* or *Syn3* (Additional file 2: Fig. S2). A two-tailed t-test showed a significant change in DNA methylation at *Syt17* by pyrosequencing at one of the two CpG sites initially identified via WGBS, however the direction of methylation change differed (Additional file 2: Fig. S2). Similarly, for *Sycp3* significant methylation changes were identified in sperm by pyrosequencing at three of the four sites identified by WGBS, but in the opposite direction (Additional file 2: Fig. S2). These genes were therefore not included in subsequent analyses.

**Fig. 3:**
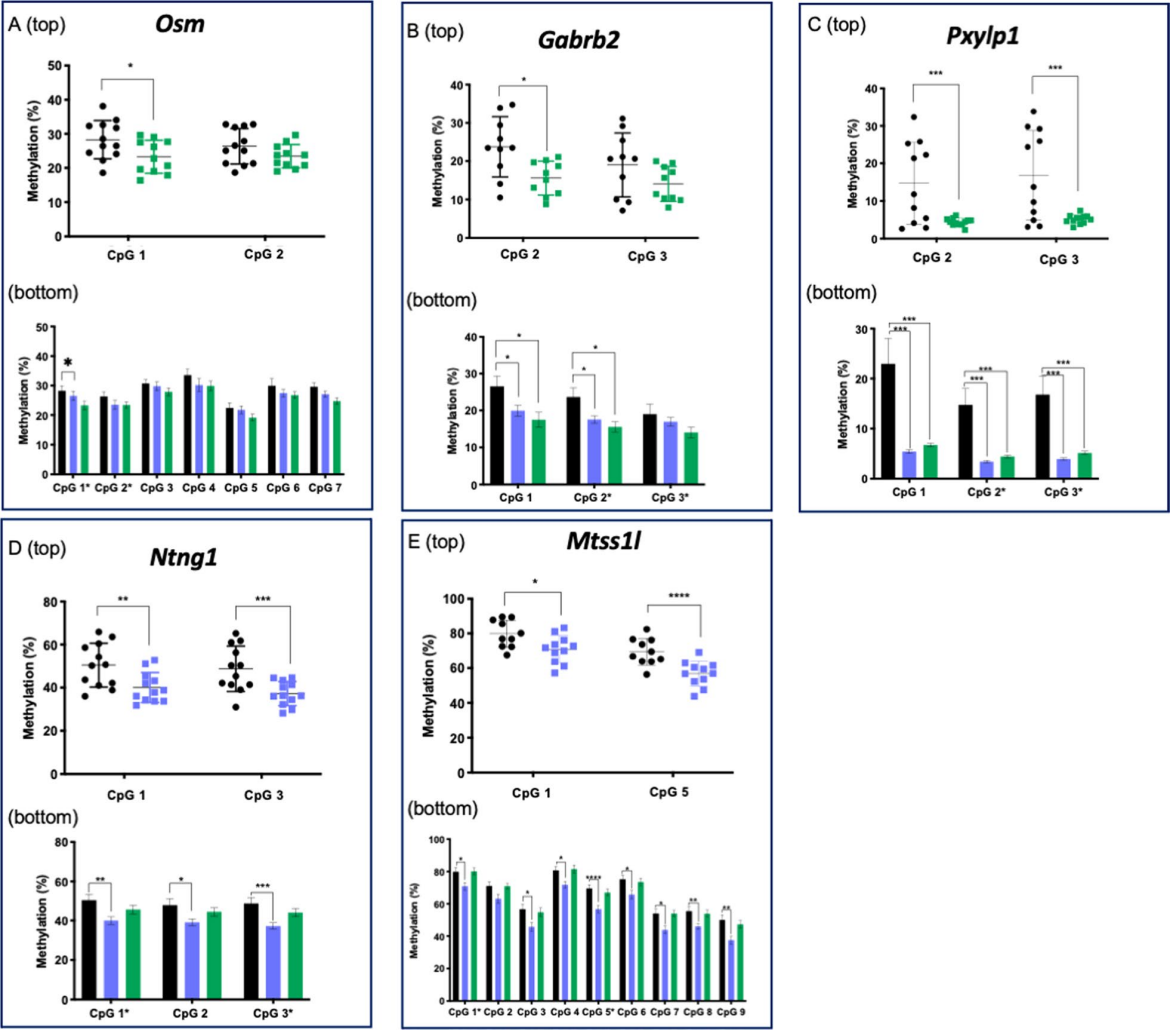
Bisulfite pyrosequencing of F0 sperm. Bisulfite pyrosequencing of CpG sites initially identified via WGBS as significantly differentially methylated between LE sperm and controls for **A** *Osm*, **B** *Gabrb2*, **C** *Pxylp1*, **D** *Ntng1*, **E** *Mtss1l*. (top) CpG sites on the x-axis correspond to those that were identified as significant by WGBS, and the measured methylation level is on the y-axis. Each point represents one individual. (bottom) CpG sites are labeled on the x-axis, mean methylation levels are plotted on the y-axis ± SEM. CpG sites labeled with “*” on the x-axis correspond to those identified via WGBS that were validated in the “top” part of the panel and are labeled with the same CpG number. Black, controls; blue, EE; green,  LE. ^*^p < 0.05, ^**^ *p* < 0.01, ^***^p < 0.005

Two-tailed t-tests for *Osm, Gabrb2, Pxylp1, Ntng1, and Mtss1l*, confirmed significant methylation changes relative to controls by pyrosequencing, in the same direction as the WGBS for the LE (Fig. 3A-E top, *p* < 0.05–0.005). We then broadened our analysis to assess methylation at all the CpG sites captured by the pyrosequencing assay to see if CE exposure similarly affected neighboring sites. We included sperm from both exposure groups to determine how the timing of the exposure impacted DNA methylation changes. A two-factor ANOVA—one factor being exposure status and one being CpG site—showed a significant main effect of exposure for *Osm, Gabrb2, Pxylp1, Ntng1,* and *Mtss1l* (*p* < 0.0001 for all genes**,** Fig. 3A-E bottom). Post hoc tests revealed no additional significant methylation changes for *Osm*.

For *Gabrb2,* in addition to validating WGBS methylation changes at CpG site 2, we detected significant hypomethylation at CpG site 1 in sperm from the LE animals relative to the controls (*p* < 0.05). At CpG sites 1 and 2, there were significant methylation differences between control and EE sperm, though the magnitude was less than that of the control and LE sperm (*p* < 0.05). For *Pxylp1*, posthoc tests were significant at all CpG sites and in both exposure groups (*p* < 0.005). For *Ntng1*, post hoc tests confirmed significant methylation changes across all three CpG sites analyzed (*p* < 0.05–0.005), but only for the EE group. There was no significant methylation difference between LE and control sperm for *Ntng1*. Lastly, for *Mtss1l*, post hoc tests revealed significant loss of methylation present in the sperm of the EE group at eight of the nine CpG sites analyzed by pyrosequencing (*p* < 0.05–0.001), but no significant changes in the LE group.

### Heritability of cannabis-induced sperm methylation changes

**Fig. 4:**
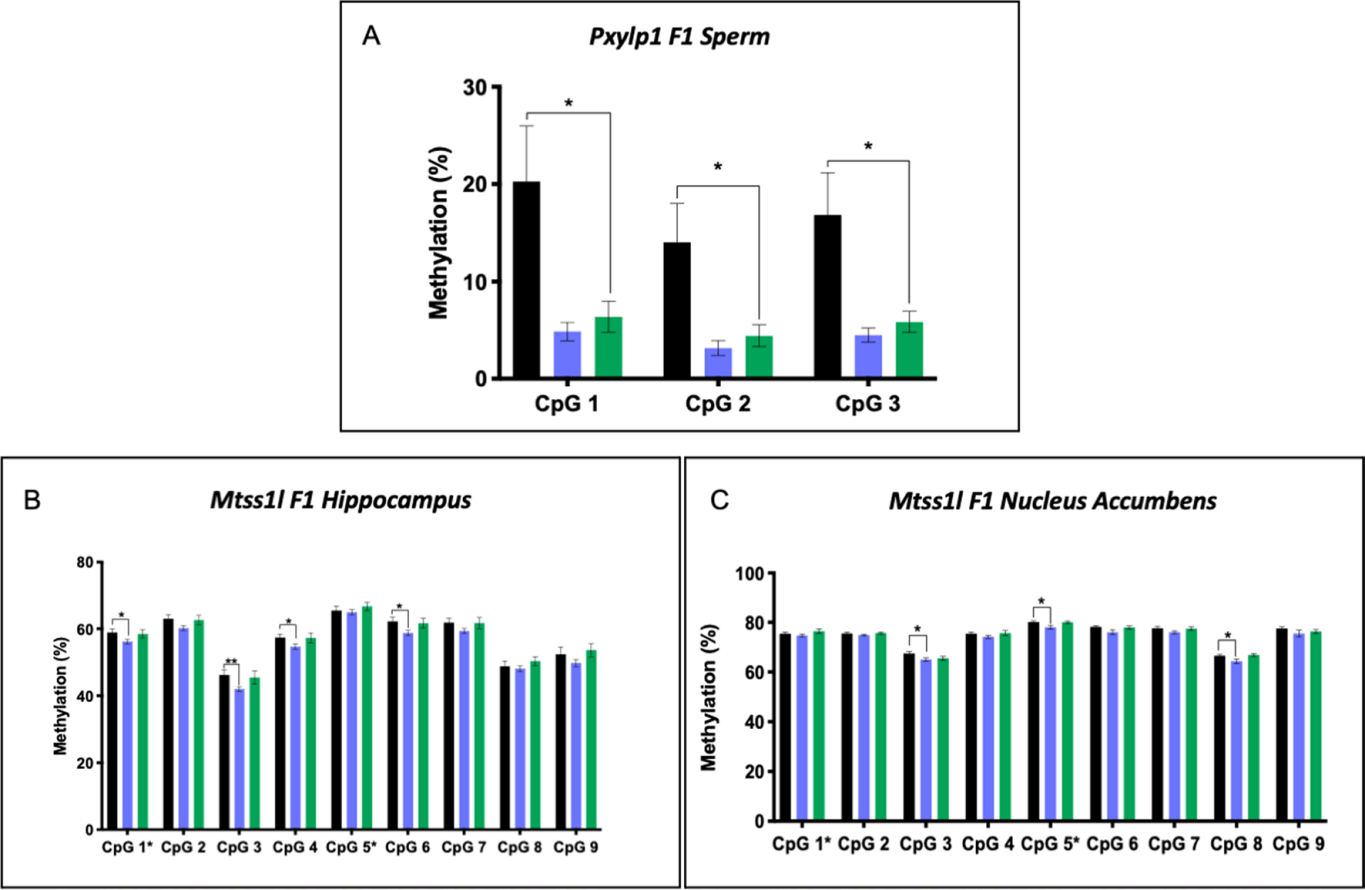
Bisulfite Pyrosequencing of F1 tissues. Bisulfite pyrosequencing detected heritable changes at **A** *Pxylp1* in F1 sperm; **B** *Mtss1l* in F1 hippocampus; and **C** *Mtss1l* in F1 NAc. CpG sites labeled with “*” correspond to those identified via WGBS in the LE v C or EE v C datasets. Black,  control offspring; blue, EE offspring; green,  LE offspring. Two-factor ANOVA (factor 1 = exposure status, factor 2 = CpG site) were significant for all genes. Significance indicators on graphs represent post hoc two-tailed t-tests. ^#^*p* = 0.05, ^*^p < 0.05, ^**^p < 0.01

To determine whether methylation changes in sperm of CE-exposed males were detectable in the next generation, we analyzed F1 tissues for methylation at CpG sites that were validated by pyrosequencing. We first examined *Pxylp1* in F1 sperm by pyrosequencing of both exposure groups given the abundant levels of this gene’s protein product in the epididymis and in mature spermatids [25]. A two-factor ANOVA revealed a significant effect of exposure on DNA methylation in F1 sperm (*p* < 0.0001). Post hoc analysis revealed a significant loss of methylation in F1 sperm from animals born to LE cannabis fathers compared to controls (*p* < 0.05, Fig. 4A), and the difference approached significance when comparing the F1 sperm from EE and control offspring (*p* = 0.06).

We next analyzed changes in DNA methylation in F1 brain tissues for genes with neuronal functions. There were no significant methylation changes for *Ntng1* in the hippocampus or nucleus accumbens (NAc; data not shown)*.* For *Gabrb2*, methylation changes were not consistent with what was observed in F0 tissues (data not shown). Therefore, we did not continue analysis of this gene given that we could not attribute this methylation change to a change in paternal sperm.

We then analyzed methylation changes at *Mtss1l* in offspring hippocampus (Fig. 4B) and NAc (Fig. 4C) tissues. We observed a significant effect of paternal exposure on DNA methylation in both F1 tissues (*p* < 0.0001). Like the methylation changes present at these CpG sites in F0 sperm, post hoc tests revealed significant losses of methylation present in EE offspring relative to control offspring. In the hippocampus, four of the nine CpG sites analyzed were significantly hypomethylated relative to controls in the EE group (*p* < 0.05–0.01). This included one of the CpG sites (CpG site 1) that was initially identified via WGBS as being hypomethylated in the EE paternal sperm. In the NAc, EE offspring were hypomethylated relative to controls for four of the nine CpG sites analyzed (*p* < 0.05). This included the other CpG site that was initially identified via WGBS (CpG site 5) as being hypomethylated in the EE paternal sperm.

### Relationships between DNA methylation and gene expression

Our finding that there are heritable changes in DNA methylation for *Mtss1l* in brain tissues prompted us to investigate whether those methylation changes are functionally related to changes in gene expression. We first examined this relationship in hippocampal tissue of the EE and control offspring at *Mtss1l* CpG site 1—one of the two sites initially identified via WGBS as being affected in paternal sperm. Pearson correlations showed no significant relationship between DNA methylation and gene expression for control or exposed offspring (Additional file 3: Fig. S3A). We stratified this analysis by sex (Additional file 3: Fig. S3B and S3C) given the known sex differences in hippocampal function [26, 27] and still observed no significant relationships between DNA methylation and gene expression.

**Fig. 5:**
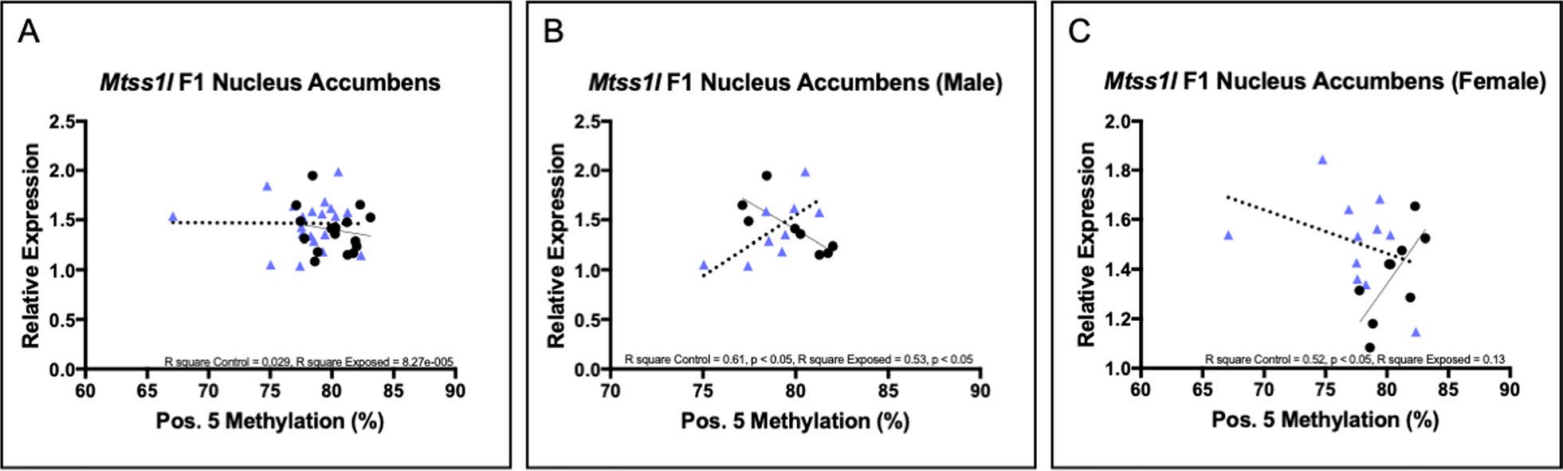
Relationship between DNA methylation at CpG site 5 and gene expression for *Mtss1l* in the NAc. Relationship between DNA methylation and gene expression for *Mtss1l* in **A** all sexes combined; **B** males only; and **C** females only. Pearson correlation R square and *p*-values are reported. Control offspring,  black circles with solid regression line; EE offspring,  blue triangles with dotted regression line

In the NAc, we examined the relationship between DNA methylation at CpG site 5 (the second site initially identified via WGBS in sperm) and gene expression for the control and EE offspring. There were no significant relationships between methylation and expression when both sexes were analyzed together (Fig. 5A). However, known sex differences in the NAc [28, 29] led us to again stratify the analysis by sex. In males (Fig. 5B), control offspring showed a significant inverse methylation-expression relationship (*p* < 0.05, R^2^ = 0.61), while exposed offspring had a significant positive methylation-expression relationship (*p* < 0.05, R^2^ = 0.53). In females (Fig. 5C), control offspring showed a significant positive methylation-expression relationship (*p* < 0.05, R^2^ = 0.52), while exposed offspring showed an inverse though non-significant relationship.

### Paternal CE exposure induces cardiomegaly in his offspring

**Fig. 6:**
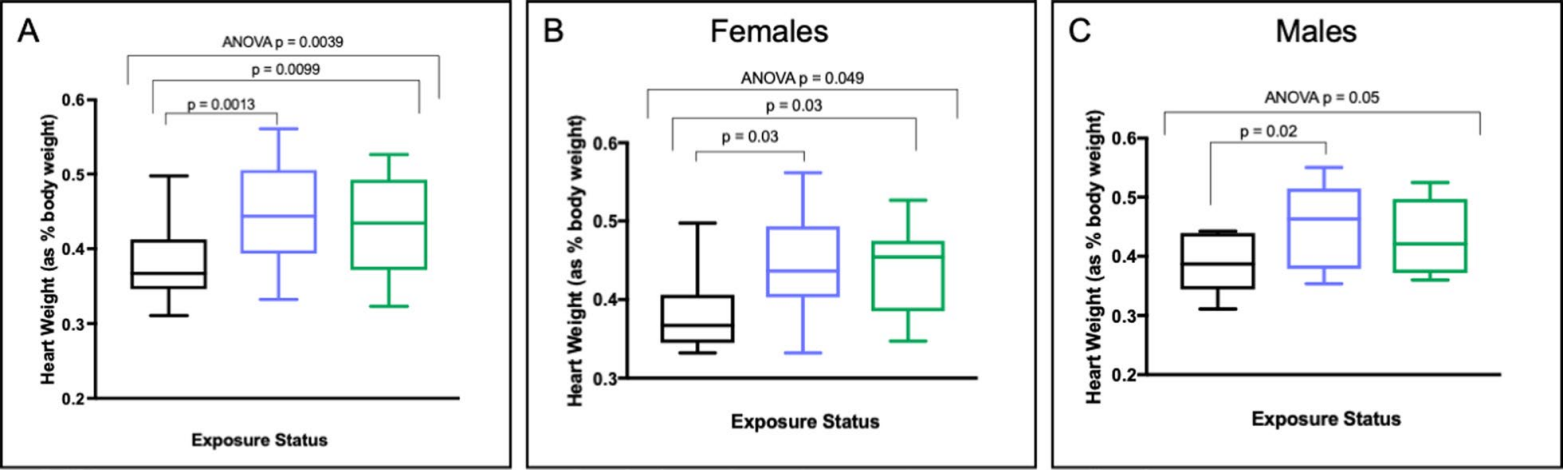
Paternal preconception exposure to CE is associated with cardiomegaly in offspring. Offspring heart weight as percent bodyweight is represented on the y-axis (in grams) for **A** all animals; **B** females only; and **C** males only. Black, control offspring; blue, EE offspring; green, LE offspring. ANOVA p-value is from a one-factor ANOVA for effect of paternal exposure status on offspring heart weight. Post hoc t-test values are reported between EE and control, and between LE and control

There is growing evidence associating prenatal cannabis exposure and teratologies in babies and children and early life exposure to cannabis has been associated with cardiovascular defects in epidemiologic and animal studies [14, 30, 31]. We measured heart weights and body weights of each of the F1 offspring from the EE, LE and control fathers and normalized heart weight to body weight. One-factor ANOVA revealed a significant effect of paternal exposure on offspring heart weight (*p* = 0.0039). Post hoc tests showed significant increased heart weight relative to controls for both the EE offspring (*p* = 0.0013), and the LE offspring (*p* = 0.0099) (Fig. 6A). Based on physiological differences in cardiovascular function and disease in males and females [32, 33], we separated this analysis by sex. A one-factor ANOVA in females (Fig. 6B) showed a significant effect of paternal exposure on offspring heart weight (*p* < 0.05). Post hoc tests showed significant increases in heart weights for both EE (*p* < 0.05) and LE (*p* < 0.05) offspring. In males (Fig. 6C), a one-factor ANOVA just approached significance for the effect of paternal exposure status on offspring heart weight (*p* = 0.05). Post hoc t-tests showed a significant increase in heart weight relative to controls only in the EE offspring (*p* < 0.05).

## Discussion

The broad goals of the present study were to characterize the effects of paternal CE exposure on the entirety of the sperm DNA methylome in a rat model of exposure, and to determine if such effects are heritable. We did not detect significant methylation differences via WGBS after implementing a conservative Bonferroni p-value threshold. This may have been due to insufficient sample size and/or insufficient coverage. As a result, we used unadjusted p values, prioritizing based on stringent thresholds to enrich for bona fide differences.

The results showed that, regardless of the timing of the exposure, most of the methylation changes occurred in the same direction in both groups relative to the controls. We next addressed the impact of the EE-specific “washout” period on the magnitude of the methylation changes present between exposed and control animals. We found that these methylation differences were dramatically diminished in the EE animals relative to the LE animals. This supports the hypothesis that exposure cessation for the duration of at least one spermatogenic cycle is effective at minimizing many of the CE-induced epigenetic effects in sperm.

However, a distinct group of CpG sites were significantly differentially methylated relative to controls in the EE dataset that were not present in the LE dataset. These results suggest that some early exposure changes persist during the wash-out period when most other changes were minimized, and that these particular changes were specific to the early exposure period. Thus, it is possible that the spermatogonial stem cells themselves were the target of this exposure and sperm derived from these particular progenitors harbor the epigenetic modifications. Any changes that may have affected only the post-spermatogonial sperm from the early exposure (and thus might resemble changes in the late exposed sperm) might no longer have been present at the time of WGBS given the resorption of sperm over the 56-day wash-out period. Importantly, if this is true, then the persistent EE-associated changes are possibly permanent, as the progenitor pool undergoes self-renewal throughout the reproductive life of a male [34]. Different epigenetic architectures and different patterns of gene expression in these cell types and phases of spermatogenesis may have further resulted in distinct CpG sites being differentially methylated.

We used IPA to determine functional relevance of the genes with the dmCpG sites. We analyzed disease and function annotations associated with the input gene lists for both datasets. There were some notable commonalities across the LE and EE analyses, including “nervous system development, function, and disease”, “developmental disorder”, and “embryonic development”. There were also notable differences. In the LE results, “cardiovascular system development, function and disease” was among the top annotations, while for the EE results there were multiple annotations associated with behavior, including spatial learning, exploratory behavior, and cognition, as well as functions associated with embryonic development including development of sensory organ, formation of eye, and lens formation.

It is interesting that, regardless of the timing of the CE exposure, there were shared functions of the genes that were significantly differentially methylated. That we saw significant terms associated with early developmental processes supports our previous findings from studies assessing the effect of THC exposure on sperm DNA methylation [18]. In that study, we found significant KEGG pathway terms associated with early developmental processes including the hippo signaling pathway. Further, we previously provided initial evidence in support of the hypothesis that genes involved in early developmental processes are increasingly sensitive to environmentally induced epigenetic disruptions [35]. The IPA findings from this study provide additional evidence in support of this hypothesis.

We next sought to validate methylation changes at select genes identified via WGBS using bisulfite pyrosequencing. At those genes that did validate WGBS findings, we asked whether methylation changes were heritable. Whether DNA methylation changes in sperm are heritable and detectable in the next generation is one of the most critical and yet incredibly challenging questions to address. We assume that a subset of sperm is randomly impacted by preconception cannabis exposure, since methylation changes in the sperm population are not universal at any given CpG site. For detection in the next generation, one of the affected sperm must successfully fertilize the egg. Further, the methylation change(s) would need to resist post-fertilization reprogramming to persist in the developing embryo where the change is then expected to be propagated across the three germ layers. This also requires that the exposure-related methylation change does not impede the sperm’s ability to successfully fertilize an egg or the viability of the embryo in order to be detectable. Of particular relevance, studies from De Domenico [36] and Innocenzi [37] have demonstrated that in vivo exposure to the synthetic cannabinoid receptor agonist JWH-133, induced altered DNA methylation at paternally imprinted genes in sperm. These changes were subsequently detectable in placental tissues and were associated with placental and embryonic defects [36, 37].

We examined F1 sperm for *Pxylp1* methylation changes given the high levels of expression of this gene in the epididymis and its enhanced presence in mature spermatids [25]. *Pxylp1* is involved in the synthesis of mature glycosaminoglycan chains and possesses hydrolase and phosphatase activity [38]. It has been associated with rare diseases such as nemaline myopathy, a hereditary congenital neuromuscular disorder characterized by muscle weakness, impaired speech abilities, and hypoventilation [39]. There was significant hypomethylation in sperm from the F1 offspring of the LE animals compared to controls*;* a methylation change that remarkably resembles that observed in the F0 LE paternal sperm. These data are consistent with intergenerational transmission of this paternal preconception exposure. It further demonstrates that the F1 generation’s sperm population is primed to pass this epigenetic alteration to the F2 generation, indicating the potential for transgenerational inheritance.

Given the involvement of *Ntng1, Gabrb2,* and *Mtss1l* in neuronal processes, we examined F1 hippocampal and NAc tissues to determine if the sperm methylation alterations are transmissible to the F1 generation. We observed changes in *Mtss1l* DNA methylation in both the F1 hippocampus and NAc comparable to the father’s sperm, consistent with intergenerational transmission of this epigenetic effect. There was an interesting temporal observation for this gene, where in both generations, only the EE fathers and their offspring exhibited methylation changes while there were no meaningful changes in the LE paternal sperm or F1 tissues. The most parsimonious explanation for these results is that the F0 spermatogonial stem cells were altered, since these changes were only evident in the exposed fathers in which there was sufficient time for the sperm derived from the spermatogonia to have been produced and matured. Methylation changes present in their offspring, led us to next assess the relationship between DNA methylation and *Mtss1l* gene expression in the F1 brains.

Analysis in the hippocampus by F0 exposure group did not reveal significant relationships between methylation and expression, and no meaningful differences were found between offspring of exposed versus control animals. However, there are sex differences in hippocampal function, which prompted us to separate male and female offspring for analysis. Control males showed a positive correlation between methylation and expression, while in the females, there was an inverse relationship. Interestingly, paternal preconception exposure to cannabis—followed by a wash-out period—caused these relationships to reverse, where male offspring now displayed an inverse relationship and females had a positive relationship between methylation and expression. These relationships were not statistically significant; however, given that *Mtss1l* plays a role in regulating synaptic plasticity, even slight methylation-mediated changes in expression could have important functional consequences. Indeed, we have previously shown that a 1% change in methylation can double or halve the level of transcription of the gene encoding IGF2 [40].

The NAc and hippocampus exhibited similar patterns of methylation-expression relationships. When we stratified analysis by sex, control males had an inverse methylation-expression relationship that was significant, while control females had a significant positive relationship. Paternal preconception cannabis exposure resulted in a significant positive *Mtss1l* methylation-expression relationship in the male EE offspring in the NAc, and an inverse relationship in the EE female offspring. This was evident despite the wash-out period following the paternal exposure.

Literature on *Mtss1l* is sparse, but recent work has focused on the role of this I-BAR domain-containing gene in regulation of plasma membrane dynamics, dendritic spine formation, and postsynaptic currents [41]. Studies have demonstrated that exercise alters expression regulation of *Mtss1l* and have identified this gene as a novel effector involved in synaptic rearrangement in an exercise-dependent manner [41]. The ability of the *Mtss1l* function to be influenced by exercise raises questions about its vulnerability to other types of influences, as we have found here with cannabis. In other studies, *Mtss1l* mutations have been associated with severe neurodegeneration and brain iron accumulation (NBIA) [42]. While this area of research is still growing, the role of epigenetics in this syndrome is not yet understood [42]. However, abundant literature suggests a role of the environment in neurodegenerative disorders, which may be mediated in part via epigenetic mechanisms [43]. There is also a role for mitochondrial dysfunction in NBIA [42]. This presents another possible avenue for future study with regard to epigenetics, since cofactors necessary for the proper establishment and maintenance of the epigenome are supplied by the mitochondria [44].

We were especially intrigued by the effect of paternal preconception CE exposure on sex-specific relationships between *Mtss1l* DNA methylation and gene expression. Increasing numbers of studies report sex differences across the epigenome, particularly in brain tissues [45–49]. Epigenetic mechanisms play important roles in maintaining these innate sexual dimorphisms, and disruption of these epigenetic regulatory states can alter brain development and sexualization [50]. Rat models of early life exposure to bisphenol A have shown altered brain sexualization [50], and preconception and perinatal exposures to multiple chemicals and drugs have sex-specific behavioral effects on offspring [51]. In fact, paternal exposure to CE results in impaired working memory in female but not male offspring, and decreased attention behaviors in males but not females [24]. Though we did not previously know if there are inherent epigenetically regulated sexual dimorphisms in these regions of the brain that regulate *Mtss1l* expression*,* it was a surprising finding to see the relationships between methylation and expression become inverted and reflective of the normal patterns seen in the opposite sex, as a result of paternal preconception cannabis exposure.

The final endpoint that we assessed in this study was physical abnormality in F1 offspring. Studies have demonstrated an association between perinatal cannabis use and cardiovascular teratologies in offspring [14, 30, 31]. One Hawaiian study found prenatal cannabis exposure was associated with multiple cardiovascular defects in babies [30]. An epidemiologic study by Wilson et al. found that paternal cannabis use is associated with a congenital heart defect in which the positions of the aorta and pulmonary artery are interchanged in offspring [31].

We observed a significant increase in heart weights after adjustment for body weight of offspring born to fathers exposed to CE prior to mating. This effect was significant for offspring born to both EE and LE fathers for females. Surprisingly in males this phenotype was only significant in offspring born to the EE fathers. While we do not understand the mechanism, this cardiomegaly finding is in line with studies associating paternal preconception and perinatal cannabis exposures with cardiovascular defects in offspring. Future studies examining morphological, histological, and expression changes in heart tissues are needed to confirm these findings.

It is curious that our cardiovascular findings and the heritable changes in DNA methylation and gene expression were mostly in offspring of fathers exposed to CE with a 56-day wash-out period prior to mating. This raises important questions about the mechanisms underlying heritability—whether attributable to DNA methylation alone, or if other epigenetic mechanisms might also be at play. One study that might provide additional clues examined paternal preconception exposure to stress, with one week or 12 weeks between the end of the stress and the initiation of mating [52]. Authors found that offspring neurodevelopment and adult stress reactivity were impacted by paternal preconception exposure to stress when there was a 12-week recovery period following stress, compared to those animals born to mice with just a one-week recovery [52]. The authors further demonstrated that this was mediated by stress-induced changes present in the epididymal epithelial cell extracellular vesicle cargo, including noncoding RNAs, that influence sperm maturation [52]. Thus, future studies of paternal preconception exposure to cannabis should investigate the interactions between DNA methylation and other epigenetic mechanisms, as well as extracellular vesicles, and how this might influence offspring outcomes.

One additional mechanism that should be investigated in future studies is the role of the placenta in mediating the effects of paternal exposure on offspring epigenetic and phenotypic outcomes. Gene expression in the placenta is enriched for paternally expressed imprinted genes, whose methylation may be altered following a paternal exposure such as CE [53]. Given that the placenta plays critical roles in regulating fetal growth and development and that the placental methylome changes in response to environmental cues [54], the interaction between the sperm epigenome and the placental epigenome should be investigated and any downstream effects on fetal developmental processes should be studied further.

Our study has several limitations. First, sample sizes were somewhat small for our paternal exposure model—there were 12 animals per group. Second, it is possible that a 56-day wash-out period was not sufficient for the effects of the CE exposure to be fully resolved. Longer wash-out periods should be examined to determine epigenetic mitigation effects. Third, while we targeted 60X coverage for our WGBS, the effective coverage averaged 20X for our study. Thus, we may not have had sufficient depth to detect all meaningful changes or to achieve significance following Bonferroni correction. Future work is needed to investigate mechanisms underlying the observed cardiomegaly phenotype, and to determine whether any of the epigenetic and gene expression changes observed in offspring correspond to any changes in offspring neurodevelopment or behavior.

## Conclusions

In conclusion, this study was the first to characterize the effect of cannabis exposure on the entire sperm DNA methylome in rats. Our results indicate that paternal exposure to CE resulted in significant changes across the sperm DNA methylome, some of which resolved with a wash-out period, but some of which persisted. We demonstrated the ability for this exposure to impact offspring DNA methylation and gene expression at genes important for neurodevelopment. This is important as prenatal cannabis exposure has been associated with neuropsychiatric disorders, and rates of autism have increased in the U.S., particularly in states where cannabis is legal. Lastly, we identified cardiomegaly in the adult F1 offspring of male and female rats born to fathers exposed to CE prior to conception. Taken together, these results demonstrate that paternal preconception exposure to cannabis affects intergenerational outcomes. As cannabis legalization expands and consumption increases, it is imperative that we improve understanding of how exposure in one generation can shape health and disease of future generations.

## Methods

All study protocols were approved by the Institutional Animal Care and Use Committee at Duke University and were conducted in accordance with federal guidelines.

### Paternal (F0) CE exposure

Paternal exposure, mating, and rearing are described in detail in Holloway et al. [24]. Three groups (*n* = 12 each, total *n* = 36) of young adult male Sprague–Dawley rats (Charles River, Raleigh, NC, USA) were used in this experiment. Male rats were randomly assigned to treatment conditions. Sample size was determined based on preliminary and prior studies indicating that a minimum of eight replicates are needed to detect a biologically relevant behavioral effect in developing rats [22, 23]. The “early exposure” group were injected (intraperitoneal, IP) with 4 mg/kg/day THC in CE in a saline solution with 5% Tween80 (NIDA Drug Supply Program, Research Triangle Institute International, Research Triangle Park, NC, USA) for 28 consecutive days, followed by 56 days without CE exposure before mating. The “late exposure” group received 4 mg/kg/day THC in CE IP for 28 consecutive days. Controls received vehicle containing saline and 5% Tween80 prior to mating. All males were bred to drug-naïve females beginning on day 87. As the injection vials were visibly different from one another (clear vehicle vs. brown–green liquid containing CE), exposures could not be blinded. However, animal IDs were used in lieu of treatment codes for partial blinding of all data collection in vivo, and the project manager separately tracked identities so that counterbalancing within data/tissue collection could be achieved. Male rats were housed 2–3 per cage with others of the same treatment group [24]. At the time of mating, male rats were paired with randomly assigned females rats. A complete description of mating and rearing is described in detail in Holloway et al. [24].

### F1 animals and tissue collection

In litters with more than 10 live pups born, pups were selected for inclusion based on the availability of male and female offspring. If either sex had *n* = 5 or less available, all were included. If the total offspring number exceeded 10, the excess pups were randomly selected for culling (e.g., 10 male, 5 female, resulted in 5 males randomly selected and culled). At weaning, one male and one female from each litter were randomly selected for behavioral testing reported in Holloway et al. [24], and the remainder were randomly selected for tissue collection at the time of each collection (*n* = 20 control offspring, *n* = 21 early exposed offspring, and *n* = 22 late exposed offspring). Body weights were recorded, and F1 offspring were sacrificed via decapitation. A complete description of maternal–fetal outcomes including litter size, anogenital distance, sex ratios, pregnancy success rates, birth weight, and weaning weight is provided in Holloway et al. [24]. Mature motile sperm were collected in PBS via the epididymal swim out method as described in our previous work [55]. The hippocampus was dissected and the nucleus accumbens (NAc) was isolated with a 2-mm-diameter punch taken from a slice encompassing this region. For offspring (*n* = 63), heart weights were recorded. All tissues were flash frozen in liquid nitrogen and were stored at − 80 °C until required.

### Nucleic acid isolation

DNA was extracted from sperm using Qiagen’s Puregene DNA Purification Protocol (Qiagen, Germantown, MD), eluted in 30 μL of nuclease-free water and stored at − 20 °C for subsequent studies. DNA and RNA were simultaneously extracted from hippocampal and NAc tissues using the Qiagen All Prep DNA/RNA Mini Kit (Qiagen). DNA was eluted in 50-100 μL of buffer TE, while RNA was eluted in 35-50 μL of nuclease-free water. DNA was stored at − 20 °C and RNA was stored at − 80 °C for further use.

### Whole genome bisulfite sequencing

*Library construction and sequencing.* For each sample, DNA concentration was measured using a Qubit (Thermo Fisher, Waltham, MA) and 250 ng was provided to the Duke Genomic and Computational Biology’s Sequencing and Genomic Technologies Core for library construction and sequencing. The DNA was fragmented using a Covaris E210 instrument (Covaris, Woburn, MA) to generate ~ 300-bp DNA fragments. Fragmented samples were converted into Illumina libraries using the Kapa HyperPrep kit (Roche, Basel, Switzerland). The libraries were bisulfite modified using the EZ DNA Methylation-Lightning Kit (Zymo Research, Irvine, CA) and amplified by PCR. Libraries were pooled in equimolar ratio and underwent 150 bp paired-end sequencing on an Illumina NovaSeq 6000 sequencer using S4 flow cells.

*Read mapping and data analysis*. Raw data were processed using the fastp algorithm to trim low quality bases and adapter sequences [56]. Read-pairs that were still at least 200 nt in length were mapped to the Rnor v6 version of the rat genome using the BSMAP algorithm [57]. Amplification artifacts were removed using the ‘MarkDuplicates’ application from the Picard toolkit [58]. The ‘methRatio’ application from BSMAP was then used to call the depth and percent methylation at each CpG locus. Sites with a depth lower than 8 × or greater than 75 × within any sample were considered missing in that sample. Sites that were not present in at least 90% of the cohort were excluded from subsequent analysis. The median coverage of all sites for each rat was then scaled to the maximum median coverage of all rats. The methylation percentage for all sites was then re-scaled accordingly and used for principal components analysis and hierarchical clustering with a correlation distance and complete linkage. RADMeth, which employs a beta-binomial regression framework, was used to test the association of each CpG site with the treatment factor pair-wise [59].

### Bioinformatic analyses

The top 10K differentially methylated CpG sites were identified for the LE and EE groups relative to controls. Each CpG site was mapped to the nearest gene. Ingenuity Pathway Analysis (IPA) software (Qiagen Digital Insights; Redwood City, CA) was used to perform analysis of top CpG sites identified from these lists with a *p*-value cutoff of 5.0 × 10^–5^ and a ≥ 10% methylation difference threshold implemented to increase stringency. Gene lists were entered into the IPA platform and top diseases and functions associated with these genes were identified.

### Bisulfite pyrosequencing

Pyrosequencing assay design was performed using the PyroMark CpG Assay Design Software (Qiagen) and sequencing was carried out using the Pyromark Q96 MD Pyrosequencing Instrument (Qiagen). PCR amplification and pyrosequencing were performed as previously described [60]. Briefly, 20 ng of bisulfite modified DNA was used as a template for PCR amplification using PCR assays that were optimized to produce a single robust band as visualized by agarose gel electrophoresis. Primers and PCR conditions used can be found in Additional file 4: Table S1. Assay validation used 0%, 25%, 50%, 75%, and 100% methylated DNAs generated by mixing fully methylated and fully unmethylated DNAs (Epitect Control DNAs; Qiagen) to demonstrate linearity in detection of increasing amounts of methylation. Once validated, samples were analyzed using optimized assay conditions.

### Quantitative real-time RT-PCR

Quantitative real-time RT-PCR was performed for *Mtss1l* and *Gabrb2* expression in F1 hippocampal and NAc brain tissues using the QuantStudio 6 Flex Real-Time PCR System from Thermo Fisher Scientific (Waltham, MA). *Mtss1l* (Taqman probe ID: Rn01432477_m1) was multiplexed 1:1 with a *Gapdh* loading control (Taqman probe ID: Rn01749022_g1) probe for each sample. PCR mastermix contained a final volume of 50 ng/μL RNA, 1 μL of each probe, 10 μl of Quantabio qScript one-step RT-qPCR ToughMix (QuantaBio, Beverly, MA), and was brought to a total reaction volume of 20 μL with nuclease-free water. Samples were run in duplicate. Cycling conditions were as follows: 50 °C for 10 min, followed by 95 °C for 1 min, followed by 40 cycles of 95 °C for 10 s and 60 °C for 1-min, with a final 1-min incubation at 60 °C.

### Statistical analysis

Statistical analyses used Prism Version 9 (GraphPad Software, San Diego, CA). Linear regression was used to determine the correlation between methylation changes in the EE and LE groups. To examine methylation changes over time, a two-tailed t-test was used with Bonferroni correction for the number of CpG sites analyzed. For bisulfite pyrosequencing aimed at confirming the WGBS findings, a two-tailed t-test was used for each CpG site, comparing the means of the exposed to control rats. For analysis of the pyrosequencing region with both CE exposure groups, we used two-factor ANOVA, with one factor being paternal exposure status and one factor being CpG site. For genes with a significant effect of exposure, a post hoc two-tailed t-test was run. Delta CT values were used to determine relative expression. Pearson correlation was run to determine relationships between DNA methylation and fold-change in expression. This analysis was run for all samples together, and then was calculated for males and females independently for each gene in each tissue due to an a priori hypothesis of sex differences in the hippocampus and NAc. Relative expression values (normalized to *Gapdh*) were multiplied by a constant value of 10 for the purposes of data visualization. For heart weight analyses, a one-factor ANOVA was run to examine the effect of paternal CE exposure on offspring heart weight. Post hoc two-tailed t-tests were run when ANOVA was significant**.**

## Supplementary Information

**Additional file 1: Figure S1.** Pyrosequencing Validation Curves. Defined mixtures of bisulfite modified fully methylated and unmethylated rat genomic DNAs were analyzed for linearity in ability to detect increasing amounts of methylation. X-axis, the input (expected) level of methylation, y-axis, the measured level of methylation. R^2^ and *p*-values values are indicated.

**Additional file 2: Figure S2.** Pyrosequencing validation of WGBS CpG sites. Bisulfite pyrosequencing of CpG sites initially identified via WGBS as significantly differentially methylated between late exposed sperm and controls for **A** *Hoxb9*; **B** *Mettl11b*; **C** *Sycp3;* **D** *Cit;* **E** *Slit2*; **F** *Lrp1*; **G** *Cit;* and early exposed and controls **H** *Grin2a;* **I** *Syn3*. CpG sites are identified as A-D on the x-axis, and the recorded methylation level is on the y-axis. Each point is an individual sperm. Black = controls, green = late exposed, blue = early exposed. ^*^p < 0.05.

**Additional file 3: Figure S3.** Relationship between DNA methylation at CpG site 1 and gene expression for *Mtss1l* in the hippocampus. Relationship between DNA methylation and gene expression for *Mtss1l* in **A** all sexes combined; **B** males only; and **C** females only. Pearson correlation R square and p-values are reported. Control offspring = black with solid regression line; early exposed offspring = blue with dotted regression line.

**Additional file 4: Table S1.** Pyrosequencing assay designs, primers, thermocycler conditions and genomic coordinates.

## Data Availability

Data are available upon request. WGBS data are available through the National Center for Biotechnology Information’s BioProject, project PRJNA753484.

## Abbreviations

CE: Cannabis extract
WGBS: Whole genome bisulfite sequencing
EE: Early exposed
LE: Late exposed
NAc: Nucleus accumbens
dmCpG: Differentially methylated CpG
