# Supplementary material for: Sperm DNA methylation alterations from cannabis extract exposure are evident in offspring
Source: Epigenetics Chromatin. 2022 Sep 10;15:33. doi: 10.1186/s13072-022-00466-3 (PMC9463823; doi:10.1186/s13072-022-00466-3)
Supplement: Supplementary file 1 — Additional file 1: Figure S1. Pyrosequencing Validation Curves. Defined mixtures of bisulfite modified fully methylated and unmethylated rat genomic DNAs were analyzed for linearity in ability to detect increasing amounts of methylation. X-axis, the input (expected) level of methylation, y-axis, the measured level of methylation. R2 and p-values values are indicated. [file 13072_2022_466_MOESM1_ESM.pptx]

## Slide 1
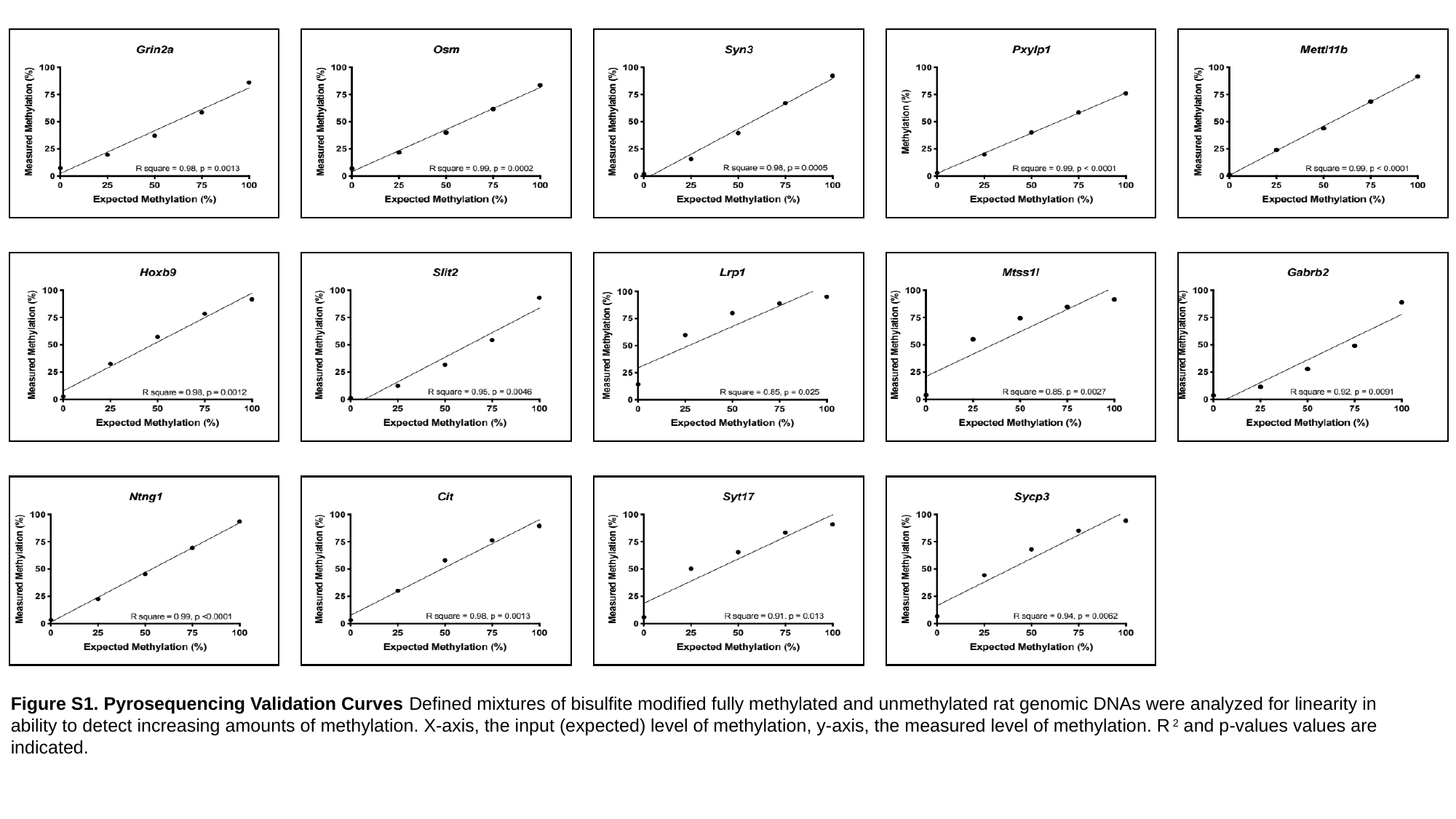

Figure S1. Pyrosequencing Validation Curves Defined mixtures of bisulfite modified fully methylated and unmethylated rat genomic DNAs were analyzed for linearity in ability to detect increasing amounts of methylation. X-axis, the input (expected) level of methylation, y-axis, the measured level of methylation. R2 and p-values values are indicated.
