# Supplementary material for: Sperm DNA methylation alterations from cannabis extract exposure are evident in offspring
Source: Epigenetics Chromatin. 2022 Sep 10;15:33. doi: 10.1186/s13072-022-00466-3 (PMC9463823; doi:10.1186/s13072-022-00466-3)
Supplement: Supplementary file 2 — Additional file 2: Figure S2. Pyrosequencing validation of WGBS CpG sites. Bisulfite pyrosequencing of CpG sites initially identified via WGBS as significantly differentially methylated between late exposed sperm and controls for A Hoxb9; B Mettl11b; C Sycp3; D Cit; E Slit2; F Lrp1; G Cit; and early exposed and controls H Grin2a; I Syn3. CpG sites are identified as A-D on the x-axis, and the recorded methylation level is on the y-axis. Each point is an individual sperm. Black = controls, green = late exposed, blue = early exposed. *p < 0.05. [file 13072_2022_466_MOESM2_ESM.pptx]

## Slide 1
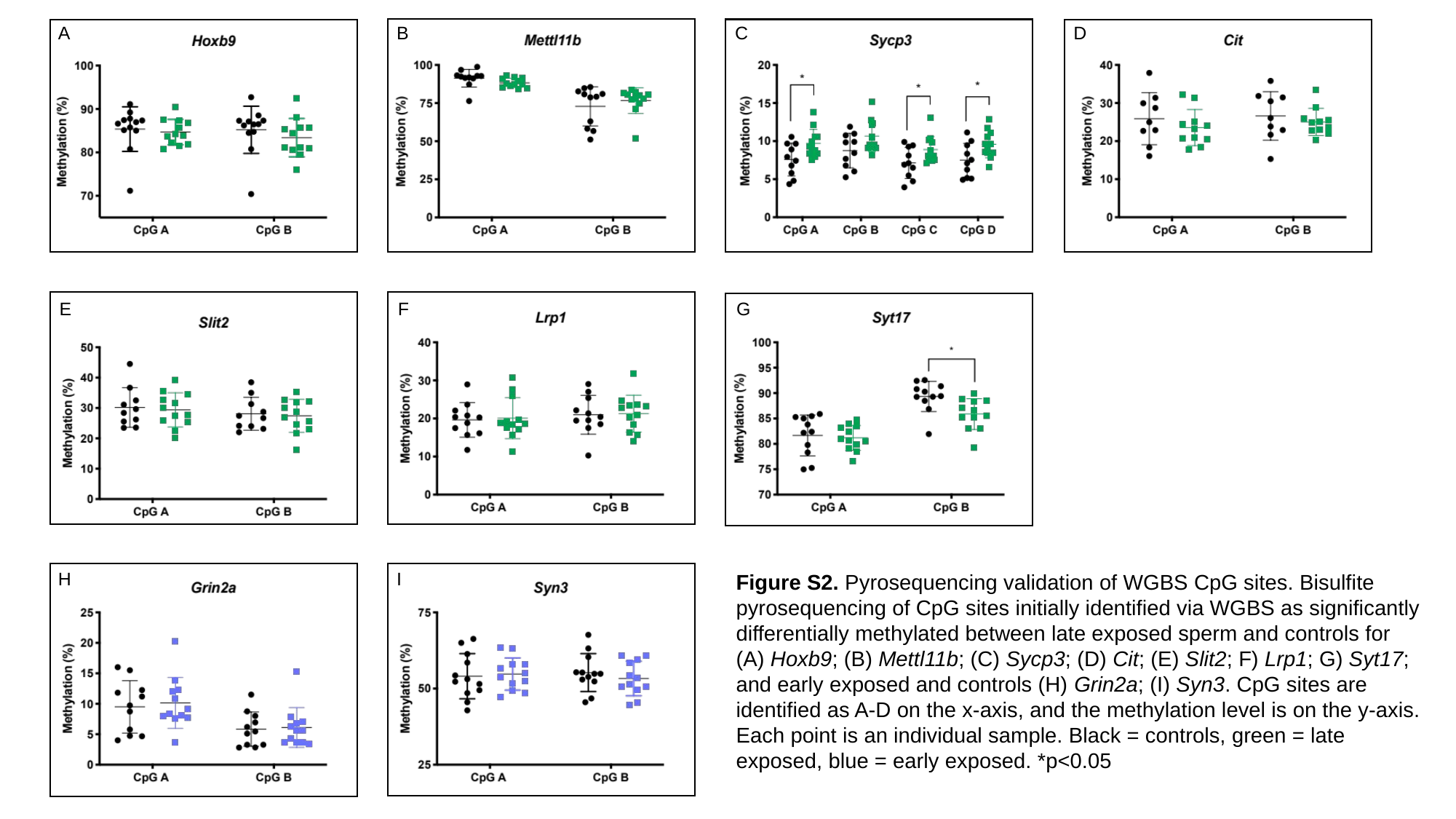

A
B
C
D
E
F
G
H
I
Figure S2. Pyrosequencing validation of WGBS CpG sites. Bisulfite pyrosequencing of CpG sites initially identified via WGBS as significantly differentially methylated between late exposed sperm and controls for (A) Hoxb9; (B) Mettl11b; (C) Sycp3; (D) Cit; (E) Slit2; F) Lrp1; G) Syt17; and early exposed and controls (H) Grin2a; (I) Syn3. CpG sites are identified as A-D on the x-axis, and the methylation level is on the y-axis. Each point is an individual sample. Black = controls, green = late exposed, blue = early exposed. *p<0.05
