# Supplementary material for: Sperm DNA methylation alterations from cannabis extract exposure are evident in offspring
Source: Epigenetics Chromatin. 2022 Sep 10;15:33. doi: 10.1186/s13072-022-00466-3 (PMC9463823; doi:10.1186/s13072-022-00466-3)
Supplement: Supplementary file 3 — Additional file 3: Figure S3. Relationship between DNA methylation at CpG site 1 and gene expression for Mtss1l in the hippocampus. Relationship between DNA methylation and gene expression for Mtss1l in A all sexes combined; B males only; and C females only. Pearson correlation R square and p-values are reported. Control offspring = black with solid regression line; early exposed offspring = blue with dotted regression line. [file 13072_2022_466_MOESM3_ESM.pptx]

## Slide 1
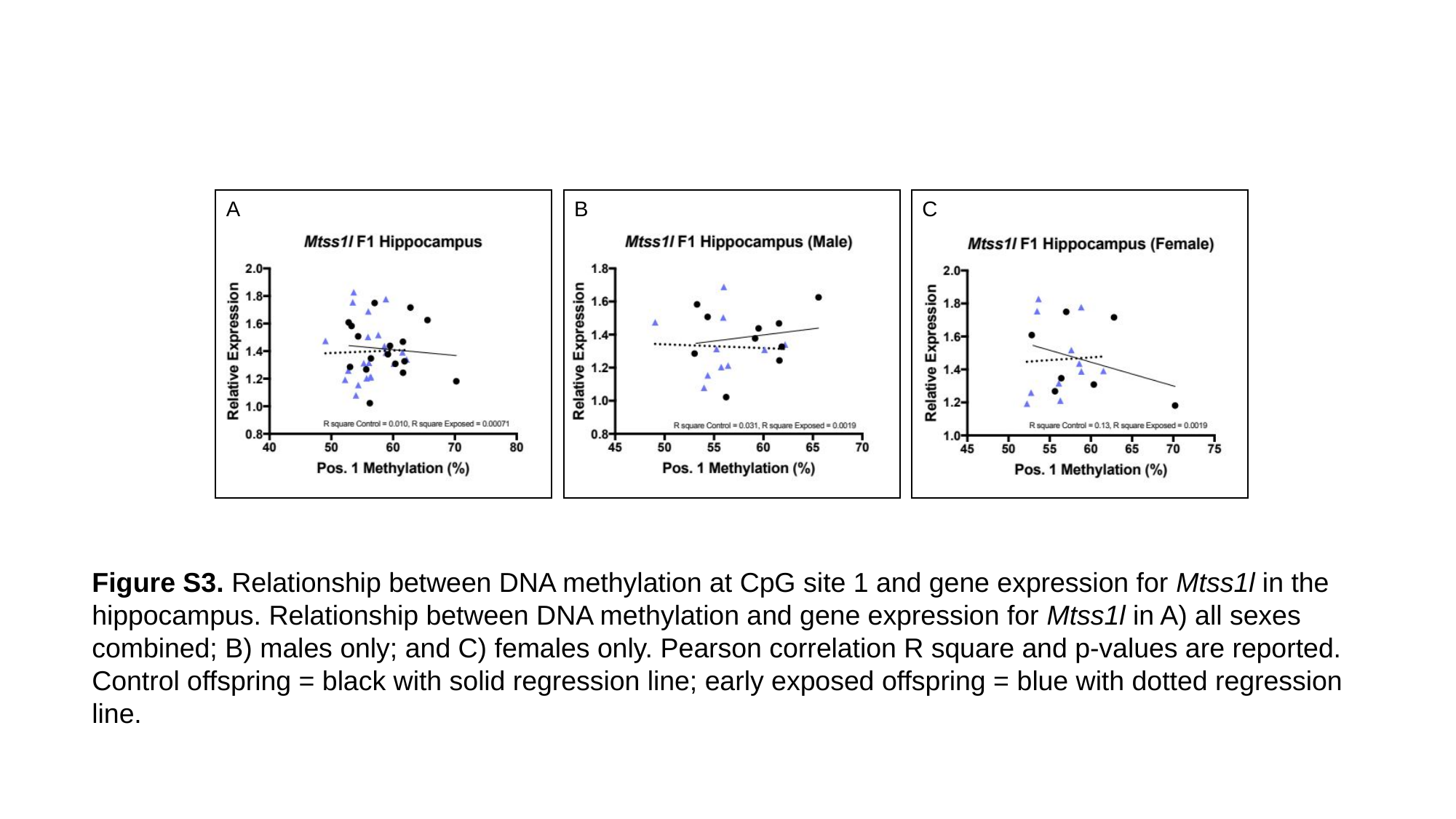

B
C
A
Figure S3. Relationship between DNA methylation at CpG site 1 and gene expression for Mtss1l in the hippocampus. Relationship between DNA methylation and gene expression for Mtss1l in A) all sexes combined; B) males only; and C) females only. Pearson correlation R square and p-values are reported. Control offspring = black with solid regression line; early exposed offspring = blue with dotted regression line.
