# Supplementary material for: Sperm DNA methylation alterations from cannabis extract exposure are evident in offspring
Source: Epigenetics Chromatin. 2022 Sep 10;15:33. doi: 10.1186/s13072-022-00466-3 (PMC9463823; doi:10.1186/s13072-022-00466-3)
Supplement: Supplementary file 4 — Additional file 4: Table S1. Pyrosequencing assay designs, primers, thermocycler conditions and genomic coordinates. [file 13072_2022_466_MOESM4_ESM.docx]

| **Gene** | **F Primer, 5'-3' (*biotin)** | **R Primer, 5'-3' (*biotin)** | **Sequencing Primer, 5'-3'** | **Sequence to Analyze, 5'-3'** | **Thermocycler Conditions** | **WGBS CpG Coordinates**  **(RGSC 6.0/rn6)** |
| --- | --- | --- | --- | --- | --- | --- |
| *Hoxb9* | AGAAGGGGAATGGATGTGAGTATA | *AAAATACATATTAAATAAAAACTCCTTCTCT | TTTAGTTAATTTTTTTGTTAATTGG | TTGTAYGTTYGTTTTTTTYGGAAAAAGYGTTGTTTTTATATTAAATATTAGAYGT | 95°C 15 min  94°C-63°C-72°C X 5\|30s  94°C-60°C-72°C X 5\|30s  94°C-57°C-72°C X 55 \|30s  72°C 10 min  4°C ∞ | Chr10:84122996  Chr10:84123018 |
| *Mettl11b* | AAGGTGAAGGAGGTTTATTTGATAATTAG | *ACCAAATCTTATCATCCAATCTCTA | GGTTTATTTGATAATTAGTAGTG | TAAYGTYGTATTGTATGAGAAGGATTYGGTATTTGGTGYGT | 95°C 15 min  94°C-65°C-72°C X 5 \|30s  94°C-62°C-72°C X 5 \|30s  94°C-59°C-72°C X 55 \|30s  72°C 10 min  4°C ∞ | Chr12:81994951  CHr12:81994983 |
| *Slit2* | *TTTTAGAGTTTGGTGGGGGAAAG | ACACTATCTAATTATTAACCCTTACCAC | CTCTAAAACTTAAATTCCCTT | TCTTCRCACAAATATTTTACTACRTTTCCATATCCCRCRAT | 95°C 15 min  94°C-65°C-72°C X 5\|30s  94°C-62°C-72°C X 5\|30s  94°C-59°C-72°C X 55\|30s  72°C 10 min  4°C ∞ | Chr14:66980552  Chr14:66980554 |
| *Lrp1* | TATTTGGGGGTAGGGTTTAGG | *TTCCTACCCCATCTCCCATTC | GTTAGTATGTGTGGGGA | YGAAGTAYGTTGTGTGYGTATYGAG | 95°C 15 min  94°C-66°C-72°C X 5 \|30s  94°C-64°C-72°C X 5 \|30s  94°C-62°C-72°C X 55 \|30s  72°C 10 min  4°C ∞ | Chr7:70853743  Chr7:70853757 |
| *Cit* | GGGGTTTGTAGTTAGTAGAGAGTAT | *AAACTCCTAAAAATTACAACCAAATATCC | GATTATTTAGATTTAGTTATTTTTG | TGTGTTTAYGTGTTTGTGTGATTTTGGGYGTGGTATGGGTAATTGYGTATGYGTGGGYGTGTYGTAG | 95°C 15 min  94°C-63°C-72°C X 5 \|30s  94°C-60°C-72°C X5 \|30s  94°C-57°C-72°C X55 \|30s  72°C 10 min  4°C ∞ | Chr12:46490405  Chr12:46490405 |
| *Syt17* | *TTGGGATAGAAGGATATATAGTTTTAAGAGTAA | ACCTCTAACCCATACTACATCACTTAA | AAAACATACAAACCTACAC | CRTTACCATATAAAAACRCTACTAAAAACRAAC | 95°C 15 min  94°C \|30s  61°C \|30s X55  72°C \|30s  72°C 10 min  4°C ∞ | Chr1:188090584  Chr1:188090584 |
| *Sycp3* | AGTTTGGGTTTTAGGAAGAGG | *ACTCCCCTCAATCCCTACCTA | TTTGGTTAGGGAGGAA | GGYGTTATAGTAGTGTTAGGTTAGATYGTTTYGTYGAGGGTAGGYGGGTTATTAATTTTGGTYGG | 95°C 15 min  94°C \|30s  61°C \|30s X55  72°C \|30s  72°C 10 min  4°C ∞ | Chr7:29030076  Chr7:29030100  Chr7:29030105  Chr7:29030108 |
| *Osm* | GAGGTTAGTGTTTTTGGATTAAGG | *ACATAACCCCAAAAACCAAAATCTC | GGATTGGTTTTTGAAGATGATA | YGGATTTTTATGTTGTTTGTTTATTTTAAGAAGGTTGAGGTTGYGGTTTATATTATTTTTGGGYGTGGGGTTGATTTYGAAGAAYGAATTYGAAGAAAAYGG | 95°C 15 min  94°C-68°C-72°C X 5\|30s  94°C-65°C-72°C X 5\|30s  94°C-63°C-72°C X 55\|30s  72°C 10 min  4°C ∞ | Chr14:84465303  Chr14:84465346 |
| *Gabrb2* | GTTGGTTTTAGTAGTGATTGTAGAT | *CTTTAAATAACCACAAATAACTATAAAAATCATA | ATTTAGTTATTAGTATTTTGGAGAT | AGAGTYGTAATTTTTAGTAATTTAGTAAAATYGAAATTTTAGAAGTTTGTAATTTATTAATTAGTAAATTTTTATTTTATAAAAYGT | 95°C 15 min  94°C-63°C-72°C X 5 \|30s  94°C-60°C-72°C X 5 \|30s  94°C-57°C-72°C X 55 \|30s  72°C 10 min  4°C ∞ | Chr10:28135390  Chr10:28135443 |
| *Pxylp1* | TAGGTTAGGGTATGTTGAATAGGA | *TAAATACAAAACCCTCATCCACACTT | TTTTTGTTTTAGTAGTGGAAT | TGGTTYGTTTTTGTTTTGGGGAGTTTGTTTTTTTAGAGTTTTTTTTATTTTAAYGGTTTTTTATTTTGGTTTTGTGAGAGTTAATTTTATTYGTAT | 95°C 15 min  94°C-65°C-72°C X 5 \|30s  94°C-62°C-72°C X 5 \|30s  94°C-59°C-72°C X 55 \|30s  72°C 10 min  4°C ∞ | Chr8:104774180  Chr8:104774218 |
| *Grin2a* | AGATTTTTTGTTTAGTGTGTGGGGATAATT | *ACTCCAAATAACTTTCCTAACCTCTTAA | TTATAAATTGTTGAGTAGAAA | GTTAGTTYGTTYGTGAAAAGTYGTTGTAGTTTATTTATAGYGYGTATTTTTAGAGAAAGAGGTAYGTGGT | 95°C 15 min  94°C-66°C-72°C X 5 \|30s  94°C-64°C-72°C X 5 \|30s  94°C-62°C-72°C X 55 \|30s  72°C 10 min  4°C ∞ | Chr10:6044945  Chr10:6044988 |
| *Syn3* | *TTGTAAGGTATTGGTTATTTTATGAAGAT | ACCAACTAAAAACCCCCAATT | ACTAAAATTATATAATATTTTCCT | TCACRAAACRACTCAACTAAAATCCAAAAAAAAATACAATAATAAAACACTCRAACCAACCACRAAA | 95°C 15 min  94°C-63°C-72°C X 5 \|30s  94°C-60°C-72°C X 5 \|30s  94°C-57°C-72°C X 55 \|30s  72°C 10 min  4°C ∞ | Chr7:23494554  Chr7:23494565 |
| *Ntng1* | *TGGAAGGAATATTTTAAATTTTTTTAGGT | AATACCCAATAAAATACTCTTCTATACA | CCAATAAAATACTCTTCTATACAA | ATAATTTCTAAAACCRTATACTAAAATAAATCCTTCACRAATTTCRA | 95°C 15 min  94°C-63°C-72°C X 5 \|30s  94°C-60°C-72°C X 5 \|30s  94°C-57°C-72°C X 55 \|30s  72°C 10 min  4°C ∞ | Chr2:212852496  Chr2:212852526 |
| *Mtss1l* | *GGTATAGTTTGGTGGAGAAGT | CACAAATCAAACCACCTACTTA | ACAAAAAAAAATAAATAACACCATC | ATAAAATACRAAACRCTAACCTATCRTTAATAACRATCCTACRCAACCRAACCCCRCRTCRAATAACCACCAACATATCTTCRACCRAAAAATCRC | 95°C 15 min  94°C-60°C-72°C X 5 \|30s  94°C-58°C-72°C X 5 \|30s  94°C-56°C-72°C X 55 \|30s  72°C 10 min  4°C ∞ | Chr19:40907156  Chr19:40907189 |
